# Evaluation of Resins for Stereolithographic 3D-Printed Surgical Guides: The Response of L929 Cells and Human Gingival Fibroblasts

**DOI:** 10.1155/2017/4057612

**Published:** 2017-10-22

**Authors:** Christoph Kurzmann, Klara Janjić, Hassan Shokoohi-Tabrizi, Michael Edelmayer, Manuela Pensch, Andreas Moritz, Hermann Agis

**Affiliations:** ^1^Department of Conservative Dentistry and Periodontology, School of Dentistry, Medical University of Vienna, Vienna, Austria; ^2^Austrian Cluster for Tissue Regeneration, Vienna, Austria; ^3^Department of Oral Surgery, School of Dentistry, Medical University of Vienna, Vienna, Austria

## Abstract

Additive manufacturing is becoming increasingly important in dentistry for the production of surgical guides. The development of cost-effective desktop stereolithography (SLA) printing systems and the corresponding resins makes this novel technique accessible to dental offices and dental laboratories. The aim of the study was to reveal the response of soft tissue cells to Clear and Dental SG resins used in desktop SLA printing systems at different stages of processing. Cell activity of L929 cells and gingival fibroblasts (GF) in response to the materials was examined in indirect and direct monolayer culture models and a direct spheroid culture model based on MTT, resazurin-based toxicity assays, and live-dead staining. Overall we found that the impact of Clear and Dental SG resins on L929 and GF depends on the processing stage of the materials. Liquid Clear resin induced a stronger reduction of cell activity compared to Dental SG resin. Printing and postcuring reduced the impact on cell activity and viability. As in-house 3D printing for surgical guides is getting integrated in the digital workflow, our data suggest that careful adherence to processing guidelines—especially postcuring—is of clinical relevance.

## 1. Introduction

The ongoing digitalization of dentistry introduces major changes to the dental practice [1, 2]. From the patient chair to the dental lab digital dentistry can be integrated in each step of the workflow allowing time- and cost-effective personalized approaches. Computer-aided impressioning, computer-aided design (CAD), and computer-aided manufacturing (CAM) have revolutionized the conventional workflow from treatment planning to accurate treatment options in dentistry as well as oral and maxillofacial surgery [1–6]. Patient's comfort, efficiency in planning, precision and accuracy, nearly any degree of freedom in production, and reproducibility are some of the advantages of these novel tools [1–6]. Currently subtractive manufacturing methods as grinding and milling are predominantly used in the dental lab, but additive manufacturing is likely to outpace them in the foreseeable future due to the upcoming technological developments. Additive manufacturing summarizes a variety of different printing techniques including fused filament fabrication (FFF), stereolithography (SLA), and selective laser sintering (SLS). Nowadays cost-effective table top printing systems are introduced to the dental market which allow novel in-house approaches in dental offices and dental laboratories.

Surgical guides in the field of dentistry were already established more than 10 years ago for oral surgery applications. By transferring radiographic digital information of computed tomography (CT) or cone beam CT to surgical templates, dental implants can be positioned more precisely which can play a major role in difficult anatomical situations allowing a better prosthetic fit consequently [7]. So far, these guides have been restricted to implant dentistry. Recent case reports have shown that modern 3D-printed templates can also be successfully used in autotransplantation of teeth and guided osteotomy and root resection [8, 9]. The production of surgical guides requires biocompatible biomaterials which do not induce adverse effects when they are in contact with the tissue. Biocompatibility and the response of the oral soft tissue to materials used in conventional as well as subtractive processed dental materials are well documented, whereas the impact of resins used in desktop SLA 3D printers is currently not entirely clear [5, 10, 11]. For most resins used in SLA printers additional postcuring is required. It is unclear how the impact of resins on oral soft tissue cells changes in the different stages of manufacturing.

The aim of the study was to reveal the impact of Clear resin and Dental SG resin used in desktop SLA 3D printing on cell viability of L929 and human gingiva fibroblasts (GF) at different processing stages. The Dental SG resin is approved for dental applications. Therefore, we performed* in vitro* tests with the L929 cell line which is used for toxicity testing and GF representing a relevant cell type of the oral soft tissue [12–14]. We applied a conventional 2D cell culture model where cells were covered with the 3D-printed specimens (indirect model) or cultured on the 3D-printed material specimens (direct model) [15]. Furthermore, to mimic the* in vivo* situation closer an innovative 3D spheroid culture model was used [12, 16–19].

## 2. Materials and Methods

### 2.1. Sample Preparation

In indicated experiments, Clear (FLGPCL02) resin and Dental SG (FLDGOR01) resin (Both Formlabs Inc., Somerville, MA, USA) were used in liquid state as purchased from the manufacturer in indicated dilutions. Resin specimens of 12 mm diameter and 1 mm thickness were printed with the Form 2 SLA 3D Printer (Formlabs Inc.). For the indicated experiments postcuring after printing of the specimens was performed for 10 min using the LC-6 Light Oven (Scheu-Dental GmbH, Iserlohn, Germany). Macroscopic and scanning electron microscopy images of the specimens were taken.

### 2.2. Preparation and Cultivation of Human Gingival Fibroblasts

After tooth extraction and after informed consent was given by the donors, human GF were isolated from extracted third molars (Ethics Committee of the Medical University of Vienna, Austria) based on a previously published protocol [20, 21]. Explant cultures were done in *α*-minimal essential medium (*α*-MEM) (Invitrogen Corporation, Carlsbad, CA, USA) supplemented with 10% fetal bovine serum (FBS; LifeTech, Vienna, Austria) and antibiotics at 37°C, 5% CO_2_, and 95% atmospheric moisture. For the experiments GF were seeded at 50,000 cells/cm^2^ and incubated for 24 hours.

### 2.3. Cultivation of L929 Cells

L929 cells were cultured in *α*-MEM supplemented with 10% FBS and antibiotics at 37°C, 5% CO_2_, and 95% atmospheric moisture. For the experiments L929 cells were seeded at 50,000 cells/cm^2^ and incubated for 24 hours.

### 2.4. Dose Response Study

**Figure 1:**
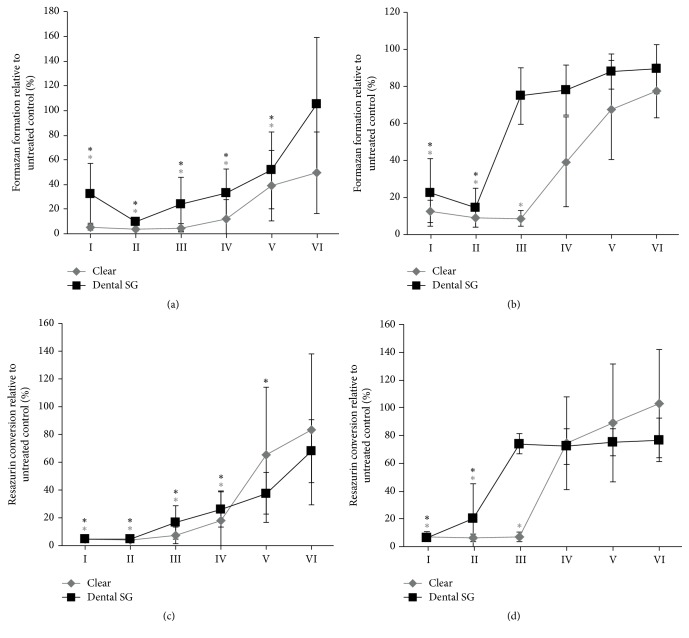
Dose response in L929 and gingival fibroblast activity to liquid Clear resin and Dental SG resin in monolayer culture model. L929 cells ((a), (c)) and gingival fibroblasts ((b), (d)) were exposed to Clear resin or Dental SG resin at 50%–0.0005%. Then cells were subjected to MTT ((a), (b)) and resazurin-based toxicity assays ((c), (d)). Data are given as mean ± standard deviation and are presented relative to the untreated control (W/O). Four independent experiments were performed. ^*∗*^*p* < 0.05, resin versus W/O; the color of *∗* indicates the specific resin; 50% (I); 5% (II); 0.5% (III); 0.05% (IV); 0.005% (V); 0.0005% (VI).

GF and L929 cells were seeded in 96-well culture plates and treated with liquid Clear resin or Dental SG resin at 50%–0.0005% of the resins for one day as indicated in Figure 1. Then cells were subjected to MTT and resazurin-based toxicity assays.

### 2.5. Indirect Culture Model

GF and L929 cells were seeded in 24-well culture plates and covered with either postcured or noncured specimens of Clear resin or Dental SG resin for one day. Then cells were subjected to MTT and resazurin-based toxicity assays. Furthermore, live-dead staining was performed.

### 2.6. Direct Culture Model

GF and L929 cells were seeded onto postcured or noncured specimens from Clear resin or Dental SG resin for one day. Then cells were subjected to MTT and resazurin-based toxicity assays. Furthermore, live-dead staining was performed.

### 2.7. Direct Spheroid Culture Model

3D GF spheroids were created using 3D Petri dishes® (Microtissues, Inc., Providence, RI, USA). Petri dishes® served as pattern to produce molds out of 2% agarose with 35 cavities for spheroids. The agarose molds were conditioned with cell culture medium and placed into 24-well plates where they were filled with cell suspensions of 75 *µ*L at 730 × 10^4^ cells/mL. Then, wells were filled with 1 mL of cell culture medium and plates were incubated for 24 hours as described above. Spheroids were cultured on cured or noncured specimens of Clear resin or Dental SG resin for one day. Afterwards spheroids were subjected to the resazurin-based toxicity assay.

### 2.8. MTT Staining

Indirect and direct cultures were incubated with 1 mg/mL MTT (3-(4,5-dimethylthiazol-2-yl)-2,5-diphenyltetrazolium bromide; Sigma-Aldrich, St. Louis, MO, USA) at 37°C for 2 hours. Formazan formation was observed under a light microscope and images were taken.

### 2.9. MTT Assay

Monolayer cultures were incubated with 1 mg/mL MTT at 37°C for 2 hours. Formazan was dissolved with DMSO. Optical density was measured using a Synergy HTX multimode reader (BioTek, Winooski, VT, USA) at a wavelength of 550 nm.

### 2.10. Resazurin-Based Toxicity Assay

A resazurin-based toxicity assay was done according to the instructions of the manufacturer. Resazurin dye solution (Sigma-Aldrich) was added in an amount equal to 10% of the culture medium and incubated at 37°C for 4 hours in the monolayer cultures (direct and indirect models) and for 8 hours in the spheroid cultures. Fluorescence was evaluated using a Synergy HTX multimode reader at a wavelength of 600 nm, using an excitation wavelength of 540 nm. The data are presented relative to the control.

### 2.11. Live-Dead Staining

Cell cultures were stained with the Live-Dead Cell Staining Kit (Enzo Life Sciences AG, Lausen, TX, USA) according to the instructions of the manufacturer. Cultures were evaluated using fluorescence microscopy for green and red, with a B-2A filter (excitation filter wavelengths: 450–490 nm), respectively. Vital cells appeared green while dead cells would have appeared red in indirect cell cultures. Images were taken. It was not feasible to take images in the direct models due to the background of the printed specimens.

### 2.12. Scanning Electron Microscopy

Scanning electron microscopy (SEM) images of the 3D-printed material were generated using the Quanta 200 system (FEI Company, Hillsboro, OR, USA). The samples were mounted on an aluminum sample holder and sputtered for the same time with a 10 nm thick gold layer using the EM ACE200 sputtering device (Leica, Wetzlar, Germany). Then images were taken from both sides of the samples at accelerating voltage of 15 kV in SE mode at 100- and 1000-fold magnification.

### 2.13. Statistical Analysis

Statistical analysis was performed with IBM SPSS Statistics Version 23 (IBM Corporation, Armonk, NY, USA), using the Kruskal-Wallis test and post hoc Mann–Whitney test. The level of significance was set at *p* < 0.05.

## 3. Results

### 3.1. Dose Response in L929 and Gingival Fibroblasts to Clear and Dental SG Resins in Monolayer Culture

To determine the effect of the polymerized resins on cell activity we treated the L929 cells and GF with the liquid resins in concentrations of 50%–0.0005% (Figure 1). We found that in both, L929 cells and GF, Clear resin and Dental SG resin reduced the levels of MTT and resazurin conversion. In L929 cells formazan formation was reduced by Clear resin and Dental SG resin at a concentration of 0.005% and higher (Figure 1(a)). Resazurin conversion was reduced by Clear resin at a concentration of 0.05% and higher and with Dental SG resin at a concentration of 0.005% and higher (Figure 1(c)). In GF formazan formation was reduced by Clear resin at 0.5% and with Dental SG resin at a concentration of 5% and higher (Figure 1(b)). Resazurin conversion was reduced by Clear resin at a concentration of 0.5% and higher and with Dental SG resin at a concentration of 5% and higher (Figure 1(d)). Overall, Clear resin in liquid form showed a slightly stronger reduction of cell activity by means of formazan and resazurin conversion compared to the Dental SG resin.

### 3.2. Activity of L929 and Gingival Fibroblasts in Response to Clear and Dental SG Resins in an Indirect Cell Culture Model

**Figure 2:**
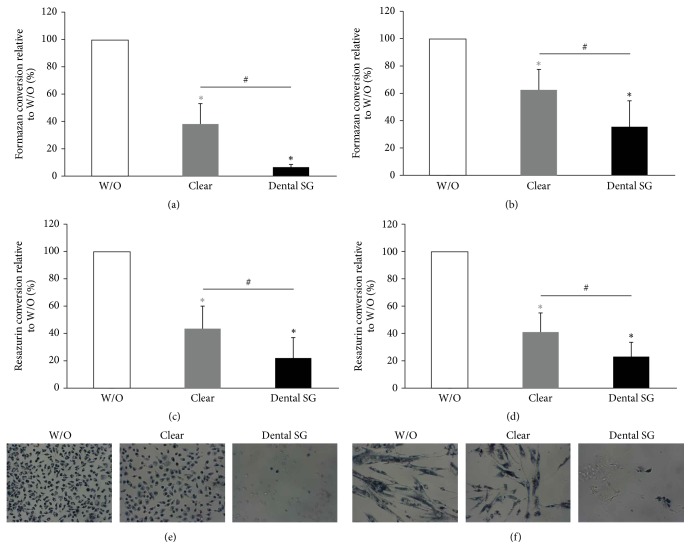
Response of L929 and gingival fibroblast activity to printed and nonpostcured Clear and Dental SG resins in an indirect cell culture model. L929 cells ((a), (c), and (e)) and gingival fibroblasts ((b), (d), and (f)) were exposed to printed Clear resin or Dental SG resin specimens which were not postcured. Then cells were subjected to MTT ((a), (b)) and resazurin-based toxicity assays ((c), (d)). Furthermore MTT staining was performed ((e), (f)). Data are given as mean ± standard deviation and are presented relative to the untreated control (W/O). Four independent experiments were performed. ^*∗*^*p* < 0.05, resin versus W/O; the color of *∗* indicates the specific resin; ^#^*p* < 0.05, Clear versus Dental SG.

**Figure 3:**
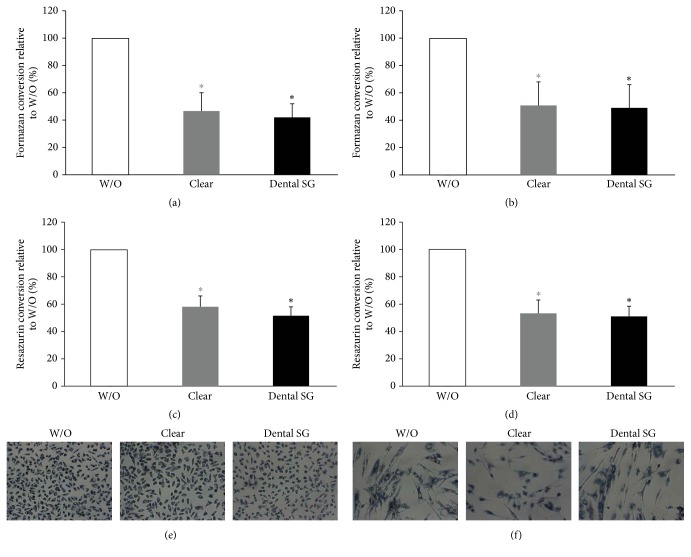
Response of L929 and gingival fibroblast activity to printed and postcured Clear resin and Dental SG resin specimens in an indirect monolayer culture model. L929 cells ((a), (c), and (e)) and gingival fibroblasts ((b), (d), and (f)) were exposed to printed and postcured Clear resin or Dental SG resin specimens. Then cells were subjected to MTT ((a), (b)) and resazurin-based toxicity assays ((c), (d)). Furthermore MTT staining was performed ((e), (f)). Data are given as mean ± standard deviation and are presented relative to the untreated control (W/O). Four independent experiments were performed. ^*∗*^*p* < 0.05, resin versus W/O; the color of *∗* indicates the specific resin.

Further, we evaluated the postcured and noncured specimens of Clear and Dental SG resins in an indirect model where the specimens were placed on monolayer cultures of L929 cells and GF. We found that formazan and resazurin conversion was reduced by specimens of all groups (Figures 2(a)–2(d) and 3(a)–3(d)). When cells were exposed to noncured printed specimens we found that Dental SG specimens showed a higher reduction in MTT and resazurin conversion compared to Clear resin (Figures 2(a)–2(d)). When evaluating the specimens that were cured after printing we found no significant difference between the impact of Clear and Dental SG specimens although both reduced MTT and resazurin conversion compared to the untreated control (Figures 3(a)–3(d)).

**Figure 4:**
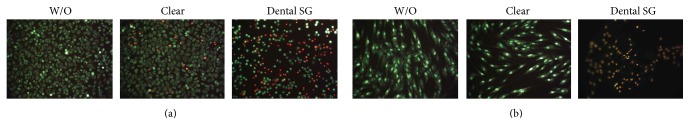
Live-dead staining of L929 and gingival fibroblasts in response to printed Clear and Dental SG resins which were not postcured in an indirect monolayer cell culture model. L929 cells (a) and gingival fibroblasts (b) were exposed to printed Clear resin or Dental SG resin specimens which were not postcured. Untreated cells served as control (W/O). The cells were subjected to live-dead staining. Living cells appeared green and dead cells appeared red.

**Figure 5:**
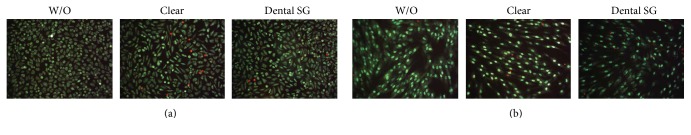
Live-dead staining of L929 and gingival fibroblasts in response to printed and postcured Clear and Dental SG resins in an indirect monolayer cell culture model. L929 cells (a) and gingival fibroblasts (b) were exposed to printed and postcured Clear resin or Dental SG resin specimens. Then cells were subjected to live-dead staining. Living cells appeared green and dead cells appeared red.

In line with this we observed reduced blue stained cells that had converted MTT to formazan in all groups that were exposed to the specimens (Figures 2(e)-2(f) and 3(e)-3(f)). However, the weakest MTT staining was observed in the L929 and GF that were exposed to the printed but not postcured Dental SG resin specimens. After curing no difference was observed between the Clear and Dental SG specimens. Similar results were observed in the live-dead staining (Figures 4 and 5). Images that had the most red cells were found in L929 and GF that were exposed to the printed but not the postcured Dental SG resin specimens (Figure 4). Again after curing no difference was observed between the Clear and Dental SG specimens (Figure 5).

### 3.3. Activity of L929 and Gingival Fibroblasts in Response to Resins in a Direct Monolayer Culture Model

**Figure 6:**
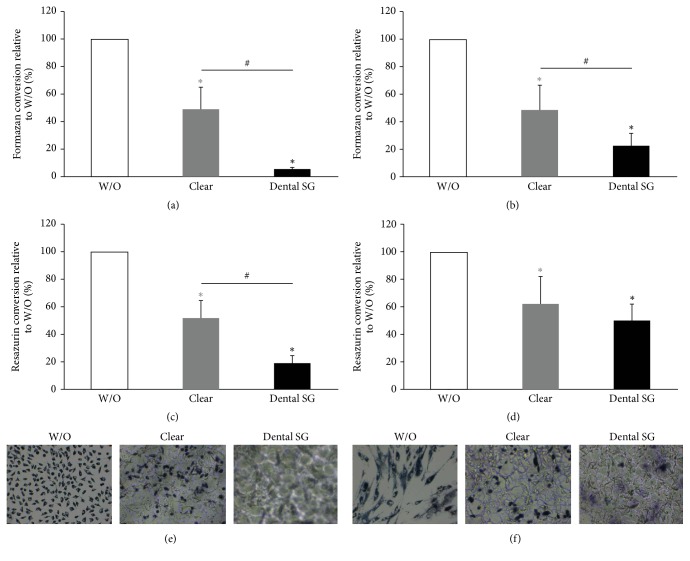
Response of L929 and gingival fibroblasts activity to printed Clear and Dental SG resins that were not postcured in a direct cell culture model. L929 cells ((a), (c), and (e)) and gingival fibroblasts ((b), (d), and (f)) were seeded and cultured on printed Clear resin or Dental SG resin specimens which were not postcured. Then cells were subjected to MTT ((a), (b)) and resazurin-based toxicity assays ((c), (d)). Furthermore MTT staining was performed ((e), (f)). Data are given as mean ± standard deviation and are presented relative to the untreated control (W/O). Four independent experiments were performed. ^*∗*^*p* < 0.05, resin versus W/O; the color of *∗* indicates the specific resin; ^#^*p* < 0.05, Clear versus Dental SG.

**Figure 7:**
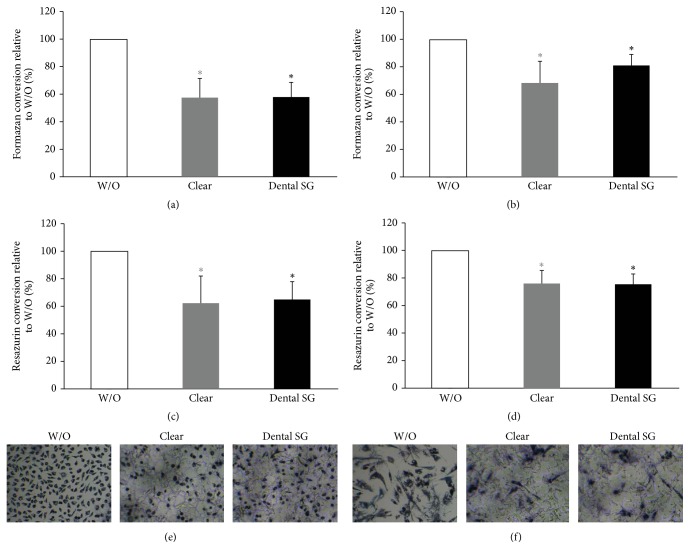
Response of L929 and gingival fibroblasts activity to printed and postcured Clear and Dental SG resin specimens in a direct monolayer culture model. L929 cells ((a), (c), and (e)) and gingival fibroblasts ((b), (d), and (f)) were seeded and cultured on printed and postcured Clear or Dental SG resin specimens. Then cells were subjected to MTT ((a), (b)) and resazurin-based toxicity assays ((c), (d)). Furthermore MTT staining was performed ((e), (f)). Data are given as mean ± standard deviation and are presented relative to the untreated control (W/O). Four independent experiments were performed. ^*∗*^*p* < 0.05, resin versus W/O; the color of *∗* indicates the specific resin.

To reveal the impact of direct contact of cells with the surface and to avoid adverse effects of the specimens due to physical pressure on the cells we used a direct approach where L929 cells and GF were seeded directly onto the specimens. Overall, the results were in line with the data of the indirect approach. We found that formazan conversion and resazurin conversion were reduced by specimens from all groups (Figures 6(a)–6(d) and 7(a)–7(d)). When cells were exposed to printed specimens which had not been cured we found that Dental SG specimens showed a higher reduction in MTT and resazurin conversion compared to Clear resin (Figures 6(a)–6(d)). When evaluating the specimens that were cured after printing we found no significant difference between the impact of Clear and Dental SG specimens although both reduced MTT and resazurin conversion compared to the control (Figures 7(a)–7(d)).

In line with this we observed a reduction in blue stained cells that had converted MTT to formazan in all groups that were exposed to the specimens (Figures 6(e), 6(f), 7(e), and 7(f)). However, the weakest MTT staining was observed in the L929 and GF that were exposed to the printed but not postcured Dental SG resin specimens. After curing no difference was observed between Clear and Dental SG specimens. However, live-dead staining could not be performed since no correct signal could be observed in this direct setup.

### 3.4. Response of Gingival Fibroblasts to Resins in a Direct Spheroid Culture Model

**Figure 8:**
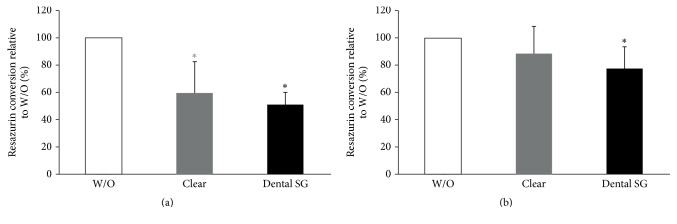
Response of gingival fibroblasts to resins in a direct spheroid culture model. Spheroids of gingival fibroblasts were seeded and cultured on printed Clear resin or Dental SG resin specimens with (b) or without (a) postcuring. Then cells were subjected to resazurin-based toxicity assays. Data are given as mean ± standard deviation and are presented relative to the untreated control (W/O). Three independent experiments were performed. ^*∗*^*p* < 0.05, resin versus W/O; the color of *∗* indicates the specific resin.

Next we used spheroid cultures to reveal the impact of direct contact in spheroid cultures. GF spheroids were seeded directly onto the specimens (Figure 8). We found that resazurin conversion was reduced by specimens of all groups. When cells were exposed to printed specimens which had not been cured we found that there was a trend showing higher reduction with the Dental SG specimens which however did not reach the level of significance (Figure 8(a)). This reduction was not so pronounced when spheroids were exposed to the cured specimens (Figure 8(b)).

### 3.5. Macroscopic and Scanning Electron Microscopic Evaluation of the Printed Specimens

**Figure 9:**
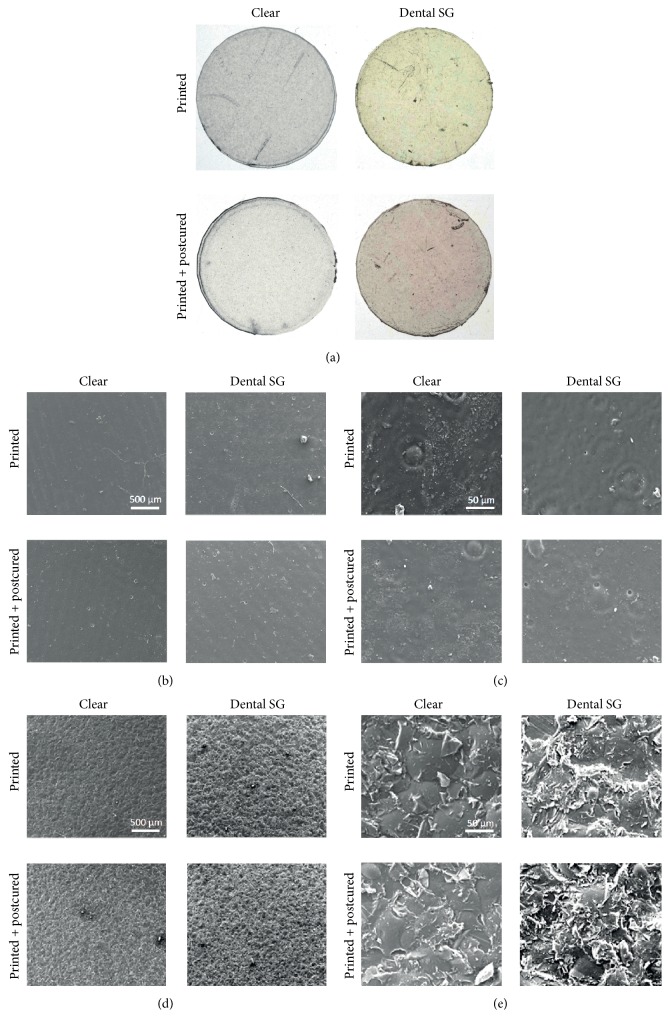
Macroscopic images and scanning electron microscopic images of the specimens. Macroscopic images of the printed specimens before and after curing (a). Scanning electron images of the specimens from the Clear and Dental SG resins with side (b, c) facing the resin tank and side (d, e) facing the printing platform. Images were taken at 100-fold magnification (b, d) and 1000-fold magnification (c, e).

Macroscopic images were taken from the 3D printed specimens from Clear resin and Dental SG resin (Figure 9(a)). We found no obvious difference between Clear resins before and after curing. Dental SG resin appeared yellow in liquid form and after printing. After curing, the color changed from yellow to orange. SEM images showed different surface topography on the side facing the building platform and the side facing the printing tank (Figures 9(b)–9(e)).

## 4. Discussion

Digitalization of dentistry together with the advances in 3D printing introduces major changes in the dental practice allowing time- and cost-efficient personalized approaches as well as higher comfort for the patients. Here we assessed the impact of two resins used in desktop SLA 3D printing: the standard Clear resin and the Dental SG resin, which is approved for dental applications. Our results show that there is a dose-dependent reduction of formazan formation and resazurin conversion in L929 and GF in monolayer cultures when exposed to the resins in liquid state. Clear resin showed a stronger impact compared to Dental SG resin, in particular in GF. When printed specimens of Clear and Dental SG resins were evaluated in indirect monolayer cultures of L929 and GF the impact on formazan formation and resazurin conversion was weaker. However, when no postcuring was performed Dental SG specimens reduced formazan formation and resazurin conversion stronger than Clear resin. This difference was abolished after postcuring. Overall, GF were not as sensitive to the impact of the resins as L929 cells.

According to the manufacturer, Clear resin contains methacrylate oligomers, methacrylate monomers, and photoinitiators [22]. The acute oral toxicity (LD50) of the components is in the range of >2000 mg/kg body weight and >2500 mg/kg body weight for methacrylated monomer and photoinitiators, respectively [22]. The acute dermal toxicity (LD50) of the components is >5000 mg/kg body weight for both [22]. Dental SG contains methacrylate oligomers and photoinitiators and has an acute oral and dermal toxicity (LD50) at >2000 mg/kg body weight. Our results are in line with these data [23, 24]. Overall, the observed reduction of cell activity based on formazan formation and resazurin conversion upon treatment with the resins in a liquid state is in line with the toxic effects of methacrylate monomers and oligomers reported in literature. These seem to be caused due to the induction of apoptosis and genotoxic effects and the delay of the cell cycle [25, 26]. Interestingly, some protective effects of antioxidants and adoptive cellular responses have been described [26, 27], suggesting that the toxicity might be related to the generation of reactive oxygen species. Our observation that the printed and postcured resins can also reduce cell activity is in line with the observations that resins are not inert but release mono- and oligomers over time [28]. It is important to choose a relevant model for the evaluation of the effect. In the indirect model the resin specimens were placed on the cell monolayers. This might lead to hypoxic conditions and physical pressure on the cells which might lead to material independent effects. To overcome these issues a direct model was chosen where cells were seeded directly onto the resin specimens to avoid the fact that samples can apply pressure to the cells. Interestingly, the results of the direct model are in line with the indirect model suggesting that the observed effects are due to the properties of the material rather than physical damage to the cells. Similar observations were reported for scaffold materials in indirect and direct models [15].

However, both the indirect and direct monolayer cultures do not directly reflect the* in vivo* situation where the surgical guides are placed onto the tissue which consists of multiple cell layers. To mimic the 3D structure of the tissue we performed spheroid cultures. This 3D culture model mimics the* in vivo* situation closer than 2D monolayer cultures [16–19]. While the generation of GF spheroids was possible, spheroids of L929 dissociated in the preliminary experiments when they were transferred onto the specimens; therefore, they could not be used for further experiments. Also here the printed specimens which were not postcured reduced the resazurin conversion. In contrast to the indirect and direct monolayer cultures no difference between the Clear and Dental SG resin was found. When printed and postcured specimens were evaluated the reduction of resazurin conversion was lower than in the specimens not postcured. These results suggest that postcuring reduces the inhibitory effect of the resin specimens on cell activity. However, we exposed the cells for 24 h to the resins in all models which does not directly reflect the* in vivo* situation where surgical guides are applied during surgery only, resulting in a shorter exposure time as presented here and thus these models might be considered more rigorous. The direct model requires that cells can attach to the surface of the specimens. Therefore we choose the 24 h frame for incubation which is in line with previous publications [15, 29]. Given the future interest in using this technology also for splints, retainers, or aligners resulting in longer exposure times, our results hold relevance in these approaches. Interestingly, the overall reduction observed in the spheroid cultures was not as pronounced as in the monolayer cultures. Based on the fact that spheroid cultures mimic the* in vivo* situation closer than monolayer cultures, future evaluations of resins for SLA 3D printing for dental applications should consider this spheroid-based approach using relevant cell types for respective research aims [12, 16–19]. Interestingly, not all cell types seem to behave similarly under these conditions.

Evaluation of the specimens on the macroscopic level showed the color change from yellow to orange as described by the manufacturer for the Dental SG resin. This change in appearance was accompanied by changes in the physical properties. No changes of the Clear resin were observed. Evaluation via SEM revealed that the surface facing the printing platform was patterned while the surface facing the printing tank showed a smooth surface. No distinct differences between the two resins were observed.

## 5. Conclusions

Overall we have shown that the impact of Clear and Dental SG resins on L929 and GF depends on the processing stages of the materials. Liquid Clear resin induces a stronger reduction of cell activity than Dental SG resin. Printing and postcuring reduced the impact on cell activity and viability. As in-house desktop 3D printing for surgical guides will be integrated in the digital workflow in the future, our data suggest that meticulous adherence to processing guidelines, especially postcuring, is of clinical relevance.

## Abbreviations

CAD: Computer-aided design
CAM: Computer-aided manufacturing
FBS: Fetal bovine serum
FFF: Fused filament fabrication
GF: Gingival fibroblasts
MTT: 3-(4,5-Dimethylthiazol-2-yl)-2,5-diphenyltetrazolium bromide
SEM: Scanning electron microscopy
SLA: Stereolithography
SLS: Selective laser sintering
*α*-MEM: *α*-Minimal essential medium.
